# Circulating Lipid- and Inflammation-Based Risk (CLIR) Score: A Promising New Model for Predicting Outcomes in Complete Colorectal Liver Metastases Resection

**DOI:** 10.1245/s10434-021-11234-0

**Published:** 2022-01-04

**Authors:** Long Bai, Xiao-Luan Yan, Yun-Xin Lu, Qi Meng, Yu-Ming Rong, Liu-Fang Ye, Zhi-Zhong Pan, Bao-Cai Xing, De-Shen Wang

**Affiliations:** 1 State Key Laboratory of Oncology in South China, Collaborative Innovation Center for Cancer Medicine, Sun Yat-sen University Cancer Center, Sun Yat-sen University, Guangzhou, 510060 People’s Republic of China; 2 Research Unit of Precision Diagnosis and Treatment for Gastrointestinal Cancer, Chinese Academy of Medical Sciences, Guangzhou, 510060 People’s Republic of China; 3 Department of VIP Region, Sun Yat-Sen University Cancer Center, Guangzhou, People’s Republic of China; 4 Key Laboratory of Carcinogenesis and Translational Research, Ministry of Education/Beijing), Beijing, 100142 People’s Republic of China; 5 Hepatopancreatobiliary Surgery Department I, Peking University Cancer Hospital and Institute, Beijing, 100142 People’s Republic of China; 6 Department of Medical Oncology, Sun Yat-sen University Cancer Center/Cancer Hospital, Guangzhou, People’s Republic of China; 7 Department of Colorectal Surgery, Sun Yat-sen University Cancer Center/Cancer Hospital, Guangzhou, Guangdong 510060 People’s Republic of China

## Abstract

**Background:**

Colorectal cancer liver metastasis (CRLM) is a determining factor affecting the survival of colorectal cancer (CRC) patients. This study aims at developing a novel prognostic stratification tool for CRLM resection.

**Methods:**

In this retrospective study, 666 CRC patients who underwent complete CRLM resection from two Chinese medical institutions between 2001 and 2016 were classified into the training (341 patients) and validation (325 patients) cohorts. The primary endpoint was overall survival (OS). Associations between clinicopathological variables, circulating lipid and inflammation biomarkers, and OS were explored. The five most significant prognostic factors were incorporated into the Circulating Lipid- and Inflammation-based Risk (CLIR) score. The predictive ability of the CLIR score and Fong’s Clinical Risk Score (CRS) was compared by time-dependent receiver operating characteristic (ROC) analysis.

**Results:**

Five independent predictors associated with worse OS were identified in the training cohort: number of CRLMs >4, maximum diameter of CRLM >4.4 cm, primary lymph node-positive, serum lactate dehydrogenase (LDH) level >250.5 U/L, and serum low-density lipoprotein-cholesterol (LDL-C)/high-density lipoprotein-cholesterol (HDL-C) ratio >2.9. These predictors were included in the CLIR score and each factor was assigned one point. Median OS for the low (score 0–1)-, intermediate (score 2–3)-, and high (score 4–5)-risk groups was 134.0 months, 39.9 months, and 18.7 months in the pooled cohort. The CLIR score outperformed the Fong score with superior discriminatory capacities for OS and RFS, both in the training and validation cohorts.

**Conclusions:**

The CLIR score demonstrated a promising ability to predict the long-term survival of CRC patients after complete hepatic resection.

**Supplementary Information:**

The online version contains supplementary material available at 10.1245/s10434-021-11234-0.

Colorectal cancer (CRC) is the third most diagnosed malignancy and the second cause of cancer-related deaths worldwide.^1,2^ Furthermore, the 5-year survival rate for CRC is only 14% for those with distant-stage disease.^3,4^ The liver is the most common and most prognostically relevant site of distant metastasis and is often the only organ involved.^5,6^ Over half of CRC patients eventually develop liver metastasis in the process of the disease.^7–9^ Although hepatic resection of colorectal liver metastasis (CRLM) offers the best chance of cure or long-term survival,^10–12^ postoperative recurrence still occurs in 70–80% of patients.^13–15^

The selection of patients likely to benefit from CRLM resection remains controversial and subjective, highlighting the urgent need to develop prognostic scoring models that can help identify different risk groups. Several prognostic models have been proposed,^16^ including the Nordlinger score,^17^ the Fong Clinical Risk Score (CRS),^18^ and the Genetic And Morphological Evaluation (GAME) score.^19^ Although broadly adopted over time, the utility of these models has been called into question in recent years.^20–23^ Although the importance of the tumor microenvironment (TME) is well recognized,^24,25^ most prior models have focused on risk factors that determine how tumor cells (‘seeds’) develop metastases and have neglected the role of the tumor environment (‘soil’). Hence, the clinical applicability of these models has been limited as they do not consider the tumor biological behavior.^26,27^

Chronic low-grade inflammation is a prevalent ongoing perturbation within the TME.^28–30^ Circulating inflammatory markers such as lymphocyte-to-neutrophil ratio (LNR), lymphocyte-to-monocyte ratio (LMR), and lactate dehydrogenase (LDH) can reflect the complex interplay between tumors and the immune state and have been gaining momentum as prognostic indicators in multiple cancer entities.^31,32^ Previous work from our group demonstrated that preoperative serum LDH levels could assist in the prognostication of curative-intent CRLM resection.^33^ LDH is a key enzyme in anaerobic glycolysis, regulated by tumor hypoxia/necrosis, and plays a pivotal role in the crosstalk between tumor and TME.^34^ By cultivating an immunodepression microenvironment, LDH can induce resistance to chemo/radio/targeted/immune therapy.^35,36^

The prevalence of obesity is rapidly increasing globally,^37^ and the understanding of the underlying links between obesity and cancer has evolved over the past decades.^38–40^ Unchecked adiposity commonly leads to chronic subclinical inflammation, a central mechanism through which adiposity promotes aggressive tumor behavior.^41,42^ Locally, adipose tissue inflammation can dramatically alter tissue compositions, thereby creating fertile soil for cancer development. Systematically, dyslipidemia, mainly comprising of raised triglyceridemia and lowered high-density lipoprotein-cholesterol (HDL-C), can sustain the inflamed microenvironment via circulating metabolic and inflammatory mediators in turn.^43–45^ In light of these findings, alterations in circulating lipid composition may reflect the TME polarization governing tumor biology. Published studies evaluating the prognostic value of lipid markers in CRC have been heterogeneous and were mainly conducted in patients with early-stage or receiving palliative systematic therapy.^44,46,47^ At the time of publication of this article, available related literature lacks evidence to link lipid metabolism with the recurrence risk of CRLM resection.

Based on these premises, we performed this two-center cohort study to systematically explore the preoperative circulating lipid and inflammation profiles in CRLM patients, aiming at developing a comprehensive prognostic scoring system for estimating the survival after complete hepatic resection.

## Methods

### Study Population

The data of consecutive CRC patients who underwent complete primary and liver metastases resection at the Sun Yat-sen University Cancer Center (SYSUCC; Guangzhou, China) and Peking University Cancer Hospital (PUCH; Beijing, China) between January 2001 and December 2016 were retrospectively assessed. Patients from SYSUCC were grouped as the training cohort and those from PUCH were grouped as the validation cohort. Detailed information, including demography, primary and metastatic tumor characteristics, pre- and postoperative treatment, blood examination, and follow-up data, were retrieved from each center’s electronic medical database.

The inclusion criteria were (1) histologically confirmed CRC; (2) evaluated as having resectable CRLM at initial diagnosis or after preoperative conversion therapy by a multidisciplinary team (MDT); (3) R0 resection of CRLM; (4) had blood biochemical examination data within 1 month before hepatectomy; and (5) were postoperatively followed up for at least 3 months. The exclusion criteria were (1) previous history of hepatectomy; (2) presence of peritoneal metastasis; (3) ablation of metastatic sites or transcatheter hepatic arterial chemoembolization (TACE) within 1 month of hepatectomy; (4) incomplete medical records; and (5) previous history of a malignant tumor.

The investigation project was examined and certified by the Ethics Committees of both centers following the Declaration of Helsinki. All patients provided written consent for the use of their data at the time of hospitalization. Since this was a non-interventional, observational, and retrospective study in which the patient data used were kept strictly confidential, informed consent was waived due to the study’s observational nature.

### Preoperative Chemotherapy

The MDT provided individualized therapeutic decisions for preoperative chemotherapy. For instance, patients with unresectable tumors or surgically unfit CRLM (approximately a Fong CRS of >2) usually received preoperative conversion therapies. The MDT evaluated treatment response to assess the feasibility of surgery.

### Blood Sample Analysis

Data from blood examination (blood routine tests, blood chemistry tests, and tumor marker tests) were eligible for analysis if performed within 1 month before hepatectomy. Each participating center’s laboratory performed the blood examination. LDH levels were classified as under or over the upper limit of normal (ULN) according to each center (250 U/L in SYSUCC and 240 U/L in PUCH). The LDL-C/HDL-C ratio (LHR) was defined as low-density lipoprotein-cholesterol divided by high-density lipoprotein-cholesterol.

### Surgery

The previously proposed definition of technical resectability mandated “a margin negative removal of all viable tumours leaving a minimum of two contiguous segments of hepatic parenchyma with adequate vascular inflow and outflow and adequate biliary drainage”.^48^ In general, patients with a normal liver can tolerate a reduction in the liver volume of up to 20%. Those with chemotherapy-induced liver injury require a future liver remnant volume of approximately 30%, and those with cirrhosis require at least a 40% residual volume.^49^ In this study, all enrolled patients completed primary tumor resection and liver metastasectomy. All the surgery specimens were confirmed by pathologic diagnosis.

### Follow-Up

Patients were followed up mainly through the outpatient clinic (or via telephone) every 3 months for the first 2 years after surgery, every 6 months for the next 3 years, and yearly after that, if there were no signs of recurrence. Physical examination, serum carcinoembryonic antigen (CEA) tests, chest computed tomography (CT) scan, and abdominal pelvic CT or magnetic resonance imaging were routinely performed at each follow-up and colonoscopy once per year after surgery. Overall survival (OS) was defined as the time from hepatic resection to death from any cause or latest follow-up, and recurrence-free survival (RFS) was defined as the time from hepatic resection to recurrence or death from any cause or latest follow-up. Subjects who were lost to follow-up or still alive at the date of the last contact were censored.

### Prognostic Scoring Model Establishment and Validation

The Circulating Lipid- and Inflammation-based Risk (CLIR) scoring model was developed in the training cohort. In order to identify independent prognostic factors, we performed univariate and multivariate Cox proportional hazards regression analyses. OS was the primary endpoint and RFS was the secondary endpoint. We explored the prognostic impacts of baseline clinicopathology variables (age, sex, hepatitis, primary tumor characteristics, *RAS/BRAF* mutation, extrahepatic disease, preoperative chemotherapy, preoperative CEA levels, characteristics of CRLM, etc.), preoperative lipid markers (body mass index [BMI], fatty liver, triglyceride levels, total cholesterol levels, LDL-C levels, HDL-C levels, and LDL-C/HDL-C levels), and preoperative inflammation markers (C-reactive protein levels and LDH levels) by the Cox regression model. Hazard ratios (HRs) and 95% confidence intervals (CIs) were calculated. Parameters with a *p* value <0.10 in the univariate analysis were selected into the multivariate analysis. Relying on the backward algorithm with a selected *p* value of 0.05, the five most significant predictors in the multivariable analysis were finally included in the CLIR score. The five variables were assigned one point for each, and total scores were defined as the CLIR score. Afterward, external validation was performed to confirm the predictive ability of CLIR score in the PUCH cohort.

Fong’s CRS was calculated as follows: number of liver metastases more than 1 (1 point); maximum diameter of liver metastases more than 5 cm (1 point); preoperative CEA level >200 ng/mL (1 point); primary lymph node-positive (1 point); and disease-free interval of fewer than 12 months after the diagnosis of primary CRC (1 point).^18^

The GAME score was calculated as follows: *KRAS* mutation (1 point); preoperative CEA level ≥20 ng/mL (1 point); primary lymph node-positive (1 point); tumor burden score (TBS) between 3 and 8 (1 point) or ≥9 (2 points); and extrahepatic disease (2 points).^19^

The models’ discriminatory ability was assessed by the area under the curve (AUC) in the time-dependent receiver operating characteristic (ROC) analysis. The accuracy of the models was further verified by Harrell’s discrimination concordance index (C-index, which is defined as the probability that predictions and outcomes are concordant) at 5 years.

### Statistical Analysis

The difference in the patient characteristics between groups was assessed by Student’s *t* tests, Mann–Whitney *U* tests, Chi-square tests, or Fisher’s exact tests, as statistically suitable. Survival curves were generated using the Kaplan–Meier method and compared using the log-rank test. For the comparison of time-dependent AUC between different models, the Wilcoxon matched-pair signed-rank test was employed.

IBM SPSS software version 20 (IBM Corporation, Armonk, NY, USA) and R software (https://www.r-project.org) were used for statistical analysis. The time-dependent AUC was calculated using the timeROC package (version 0.3; CRAN.R-project.org/package = timeROC), and the C-index was calculated using the *rms* package (version 5.1–3.1). To identify the threshold of continuous variables (such as serum biomarker levels) to discriminate patients according to OS, X-tile software (version 1.9) was applied to determine the best cut-off values.^50^ *P* values <0.05 were considered statistically significant, and two-tailed tests were used.

## Results

### Patient Characteristics

Overall, the data of 380 consecutive patients from SYSUCC and 354 patients from PUCH, treated between January 2001 and December 2016, were retrospectively assessed. Of these patients, 39 from SYSUCC and 29 from PUCH were excluded based on the following exclusion criteria: preoperative peritoneal metastasis (*n* = 12); R1 or R2 resection (*n* = 27); loss to follow-up (*n* = 11); previous history of hepatectomy (*n* = 2); ablation of metastatic sites or TACE within 4 weeks of hepatectomy (*n* = 14); and previous history of malignant tumor (*n* = 2). Finally, 341 patients from SYSUCC were included in the training cohort and 325 from PUCH were included in the validation cohort.

The demographic and clinicopathological characteristics of the patients are summarized in Table 1. All enrolled patients were Chinese. The training cohort consisted of 228 males and 113 females, with a median age of 57 years. After a median follow-up time of 62.9 months, the median OS was 63.3 months (95% CI 49.8–76.1 months); the 1-, 3-, and 5-year OS rates were 92.9%, 66.9%, and 51.7%, respectively. The median RFS was 21.1 months (95% CI 16.6–25.7 months). In the training cohort, 78 (22.9%) patients had right-sided primary tumor, 188 (55.1%) patients had primary lymph node-positive, 230 (67.4%) patients had synchronous CRLM, 34 (10.0%) patients had extrahepatic disease, 194 (53.1%) patients had multiple liver metastases, 59 (17.3%) patients had CRLM >5 cm, 176 (51.6%) patients received preoperative chemotherapy, and 240 (70.4%) patients received postoperative adjuvant chemotherapy. The proportion of patients with Fong scores of 0–1, 2–3, and 4–5 were 24.9%, 68.9%, and 5.6%, respectively.

**Table 1:** Patient clinicopathologic characteristics

Characteristics	Training cohort [*n* = 341]	Validation cohort [*n* = 325]
Median follow-up, months (95% CI)	62.9 (59.0–66.8)	76.0 (71.7–80.3)
Median OS, months (95% CI)	63.3 (44.2–81.3)	57.0 (44.1–69.9)
Median RFS, months (95% CI)	21.1 (16.6–25.7)	17.0 (0.87–33.1)
*Patient characteristics*		
Age [median (range)]	57 (26–82)	57 (24–80)
Sex		
Male	228 (66.9)	214 (65.8)
Female	113 (33.1)	111 (34.2)
Preoperative CEA, ng/mL		
> 5	129 (37.8)	105 (32.3)
≤ 5	211 (61.9)	220 (67.7)
Missing data	1 (0.7)	0 (0)
Preoperative LDH		
> ULN	36 (10.6)	35 (10.8)
≤ ULN	305 (89.4)	290 (89.2)
*Primary tumor characteristics*		
Location^a^		
Right-sided	78 (22.9)	52 (16.0)
Left-sided	263 (77.1)	273 (84.0)
Pathology		
Adenocarcinoma	318 (93.3)	318 (97.8)
Non-adenocarcinoma	21 (6.2)	7 (2.2)
Missing	2 (0.6)	0 (0)
Lymph node metastases		
Absent	153 (44.9)	110 (33.8)
Present	188 (55.1)	215 (66.2)
*CRLM characteristics*		
Maximum diameter of CRLM, cm		
≤ 5	295 (82.7)	275 (84.6)
> 5	59 (17.3)	50 (15.4)
Number of metastases		
1	167 (46.9)	140 (43.1)

Data are expressed as *n* (%) unless otherwise specified

^a^Colorectal cancer arising in or proximal to the splenic flexure was defined as right-sided, and that arising distal to the splenic flexure were defined as left-sided

*OS* overall survival, *RFS* recurrence-free survival, *CEA* carcinoembryonic antigen, *CRLM* colorectal liver metastases, *LDH* lactate dehydrogenase, *ULN* upper limit of normal, 250 U/L in the training cohort and 240 U/L in the validation cohort

The validation cohort consisted of 214 males and 111 females, with a median age of 57 years. The median follow-up time was 76.0 months and the median OS was 57.0 months (95% CI 44.1–69.9 months). The 1-, 3-, and 5-year OS rates were 96.3%, 61.4%, and 47.2%, respectively. The median RFS was 17.0 months (95% CI 0.87–33.1 months). The demographic data, tumor characteristics, and treatment patterns were well comparable between the two cohorts, except that patients in the validation cohort had a higher proportion of left-sided primary tumor (84.0% vs. 75.6%), metachronous CRLM (46.5% vs. 31.6%), primary lymph node metastases (66.2% vs. 56.0%), and preoperative chemotherapy (63.7% vs. 52.5%) than patients in the training cohort.

### Identification of Risk Factors for Overall Survival in the Training Cohort

Univariate and multivariable Cox regression analyses revealed the relationship between clinicopathology variables and OS in the training cohort (Table 2). In the univariate analysis, sex, primary lymph node metastases, pathology differentiation, preoperative chemotherapy, size of CRLMs, number of CRLMs, preoperative CEA levels, total cholesterol levels, LDL-C levels, LHRs, and LDH levels were identified as potential prognostic markers (*p* < 0.10). These variables were subsequently introduced in the multivariate analysis.

**Table 2:** Univariate and multivariate analyses for predictors of overall survival in the training cohort

Variables	Univariate analysis		Multivariate analysis	
	HR (95% CI)	*p* Value	HR (95% CI)	*p* Value
Age	1.26 (0.96–1.66)	0.104		
Female sex	0.72 (0.51–1.01)	0.057	0.75 (0.52–0.93)	0.028*
BMI	1.04 (0.98–1.10)	0.190		
Diabetes	1.52 (0.89–2.58)	0.124		
Fatty liver	0.77 (0.48–1.24)	0.289		
Hepatitis^a^	0.88 (0.50–1.56)	0.669		
Sidedness, left vs. right^b^	0.94 (0.65–1.36)	0.760		
*Pathology*				
Poor differentiation	1.40 (0.98–2.00)	0.064	1.39 (0.96–2.00)	0.078
Adenocarcinoma	0.93 (0.49–1.77)	0.834		
Primary T4 stage	1.18 (0.85–1.65)	0.328		
Node-positive primary	1.77 (1.27–2.47)	0.001	1.88 (1.35–2.61)	<0.001*
CEA levels	1.00 (0.99–1.00)	0.302		
Metachronous CRLM	0.81 (0.59–1.12)	0.202		
Number of CRLMs	1.19 (1.11–1.27)	<0.001	1.12 (1.03–1.21)	0.006*
Maximum diameter of CRLM	1.17 (1.09–1.25)	<0.001	1.17 (1.08–1.26)	<0.001*
*KRAS/NRAS/BRAF* mutated	0.96 (0.58-1.57)	0.865		
Extrahepatic disease	1.46 (0.90–2.35)	0.124		
Preoperative chemotherapy	1.77 (1.28–2.43)	0.001	1.44 (1.02–2.03)	0.039*
LDH levels	1.002 (1.001–1.003)	<0.001	1.001 (1.00–1.003)	0.026*
C-reactive protein	1.01 (1.00–1.02)	0.156		
Triglyceride levels	0.96 (0.83–1.11)	0.592		
Total cholesterol	1.15 (1.01–1.32)	0.039	1.08 (0.78–1.50)	0.646
LDL-C	1.22 (1.06–1.42)	0.007	0.91 (0.58–1.43)	0.673
HDL-C	0.84 (0.54–1.31)	0.447		
LDL-C/HDL-C	1.27 (1.08–1.50)	0.004	1.23 (1.04–1.45)	0.015*

^a^Including hepatitis A, B, C, and E

^b^Colorectal cancer arising in or proximal to the splenic flexure was defined as right-sided; arising distal to the splenic flexure was defined as left-sided

*HR* hazard ratio, *CI* confidence interval, *CEA* carcinoembryonic antigen, *CRLM* colorectal liver metastases, *BMI* body mass index, *LDH* lactate dehydrogenase, *LDL-C* low-density lipoprotein cholesterol, *HDL-C* high-density lipoprotein cholesterol, * indicates statistical significance

Finally, seven independent predictors of OS were identified: female sex (HR 0.75, 95% CI 0.51–0.93; *p* = 0.028), primary node-positive (HR 1.88, 95% CI 1.35–2.61; *p* < 0.001), number of CRLMs (HR 1.12, 95% CI 1.03–1.21; *p* =0.006), maximum diameter of CRLM (HR 1.17, 95% CI 1.08–1.26; *p* < 0.001), preoperative chemotherapy (HR 1.44, 95% CI 1.02–2.03; *p* = 0.039), preoperative serum LDH levels (HR 1.001, 95% CI 1.00–1.003; *p* = 0.026), and preoperative serum LHRs (HR 1.23, 95% CI 1.04–1.45; *p* = 0.015). Subsequently, we chose the five most significant predictors (with the lowest *p*-values) to comprise the CLIR score.

### Establishment of the Circulating Lipid- and Inflammation-Based Risk (CLIR) Score

The X-tile software was used to identify the best cut-off values of LHRs, LDH levels, number of CRLMs, and size of CRLMs. The results showed that the best cut-off value of LHR was 2.9, LDH level was 250.5 U/L, number of CRLMs was four, and size of CRLMs was 4.4 cm. Therefore, the five independent predictors of poor OS, namely primary node-positive, number of CRLMs more than four, maximum diameter of CRLMs more than 4.4 cm, preoperative LDH levels above 250.5 U/L, and LHR over 2.9 were incorporated into the CLIR score. Each factor was assigned one point and the total score (0–5) was calculated.

### Model Internal Validation: Survival Outcomes Assessed by the Fong and CLIR Scores

**Fig. 1:**
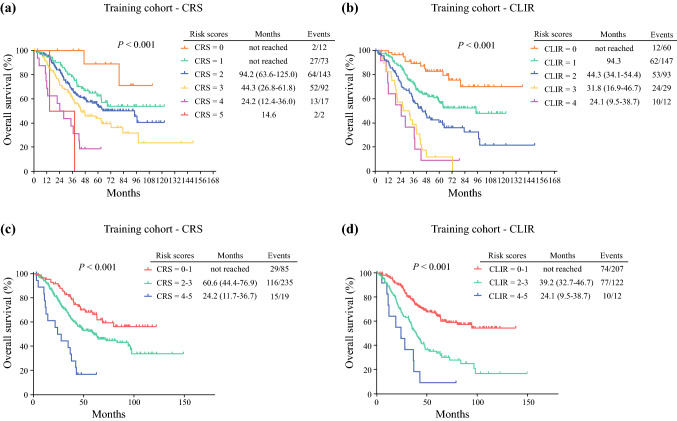
Kaplan–Meier analysis of overall survival of patients in the training cohort stratified according to different scoring systems. (**a, b**) Overall survival was stratified by different scores of CRS or CLIR in the training cohort. (**c, d**) Overall survival was stratified by different risk groups of CRS or CLIR score in the training cohort; three categories: score 0–1, score 2–3, and score 4–5. *CRS* clinical risk score, *CLIR* Circulating Lipid- and Inflammation-based Risk score

In the training cohort, OS stratified by different risk scores (0–5) as defined by CRS and CLIR is shown in Fig. 1. The survival curves were more discriminated in the CLIR model than in the CRS model. In the CRS model, the median OSs for scores 0–5 were not reached, not reached, 94.2 months, 44.3 months, 24.2 months, and 14.6 months, respectively, while in the CLIR model, the median OSs for scores 0–4 were not reached, 94.3 months, 44.3 months, 31.8 months, and 24.1 months, respectively (Figs. 1a, b).

In the Fong score, the median RFS for scores 0–5 were not reached, 31.2 months, 24.9 months, 12.2 months, 6.9 months, and 4.5 months, respectively, while in the CLIR score, the median RFS for scores 0–4 were not reached, 26.9 months, 15.1 months, 9.0 months, and 3.7 months, respectively (Fig. 3a, b).

The CRS and CLIR scores were further divided into low (0–1 points)-, intermediate (2–3 points)-, and high (4–5 points)-risk groups. In the CRS and CLIR scores, the median OSs of the high-risk group were 24.2 months and 24.1 months, respectively, and those of the intermediate-risk group were 60.6 months and 39.2 months, respectively, while those of the low-risk group were both not reached (Fig. 1c, d). In the CLIR score, the 5-year survival rates in the low-, intermediate-, and high-risk groups were 64.8%, 33.7%, and 9.2%, respectively, while in the Fong score, the 5-year survival rates were 66.3%, 49.3%, and 16.7% in the corresponding risk groups.

In the Fong score, the median RFS were 59.3 months, 18.4 months, and 6.9 months in the low-, intermediate-, and high-risk groups, respectively. In the CLIR score, the median RFS in the corresponding groups was 39.4 months, 15.2 months, and 8.3 months, respectively (electronic supplementary Fig. S1).

### Model External Validation: Survival Outcomes Assessed by the Fong and CLIR Scores

**Fig. 2:**
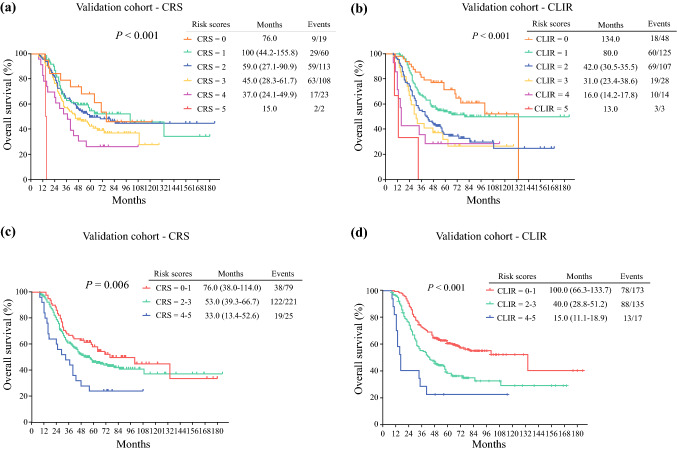
Kaplan–Meier analysis of overall survival of patients in the validation cohort stratified according to different scoring systems. (**a, b**) Overall survival was stratified by different scores of CRS or CLIR in the validation cohort. (**c, d**) Overall survival was stratified by different risk groups of CRS or CLIR score in the validation cohort; three categories: score 0–1, score 2–3, and score 4–5. *CRS* clinical risk score, *CLIR* Circulating Lipid- and Inflammation-based Risk score

**Fig. 3:**
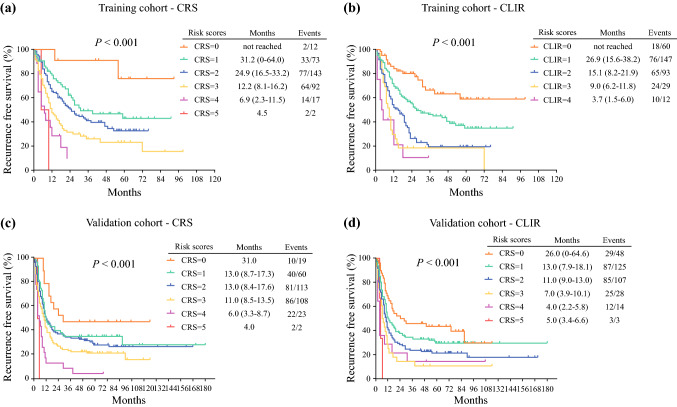
Kaplan–Meier analysis of recurrence-free survival stratified according to different scoring systems. (**a, b**) Recurrence-free survival stratified by different scores of CRS or CLIR in the training cohort. (**c, d**) Recurrence-free survival stratified by different scores of CRS or CLIR in the validation cohort. *CRS* clinical risk score, *CLIR* Circulating Lipid- and Inflammation-based Risk score

In the validation cohort, the Kaplan–Meier analysis of OS for different scores is shown in Fig. 2. The median OS in the CRS and CLIR models was score 0 (76.0 months vs. 134.0 months), score 1 (100.0 months vs. 80.0 months), score 2 (59.0 months vs. 42.0 months), score 3 (45.0 months vs. 31.0 months), score 4 (37.0 months vs. 16.0 months), and score 5 (15.0 months vs. 13.0 months) (Fig. 2a, b). The median RFS in the CRS and CLIR models was score 0 (31.0 months vs. 26.0 months), score 1 (13.0 months vs. 13.0 months), score 2 (13.0 months vs. 11.0 months), score 3 (11.0 months vs. 7.0 months), score 4 (6.0 months vs. 4.0 months), and score 5 (4.0 months vs. 5.0 months) (Fig. 3c, d).

Different risk groups were also evaluated by the two scoring systems. Median OSs of the low-risk group in the CRS and CLIR scores were 76.0 months and 100.0 months, respectively, and those of the intermediate-risk group were 53.0 months and 40.0 months, respectively, while those in the high-risk group were 33.0 months and 15.0 months, respectively (Fig. 2c, d). In the CLIR score, the 5-year survival rates in different risk groups were low-risk (58.8%), intermediate-risk (35.3%), and high risk (18.8%). In the Fong score, the 5-year survival rates in the three risk groups were 58.1%, 46.1%, and 24.0%, respectively.

In the Fong score, the median RFS of the low-, intermediate-, and high-risk groups were 17.0 months, 12.0 months, and 6.0 months, respectively. The median RFS in the corresponding groups were 17.0 months, 10.0 months, and 4.0 months in the CLIR score (electronic supplementary Fig. S1). The above results indicated that the CLIR score’s better prognostic discriminatory ability was also confirmed in the validation cohort.

### Comparison of the CLIR Score with the Fong Score in Prediction Ability

**Fig. 4:**
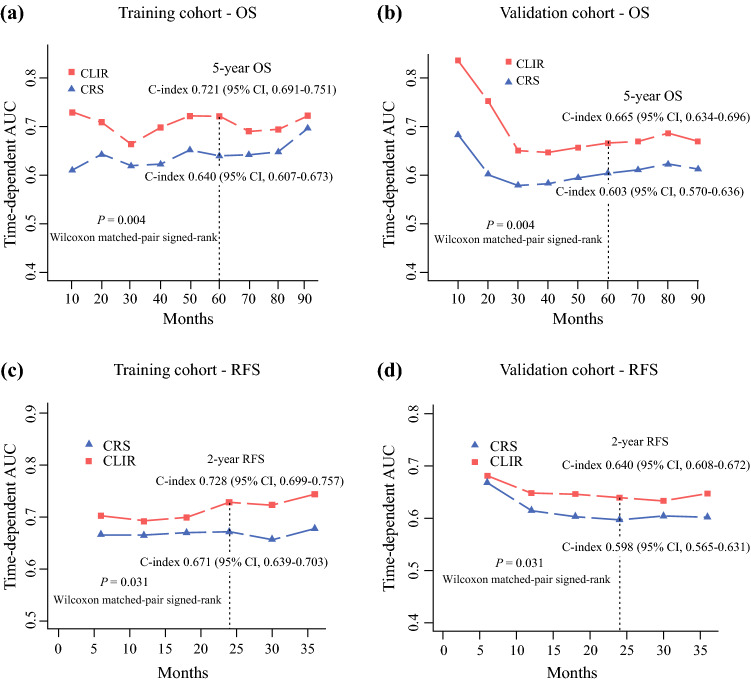
Comparison of different scoring systems in the prediction of overall survival. (**a**) Time-dependent AUCs of the CRS and CLIR score in the training cohort. (**b**) Time-dependent AUCs of the CRS and CLIR score in the validation cohort. *CRS* clinical risk score, *CLIR* Circulating Lipid- and Inflammation-based Risk score, *AUC* area under curve, *OS* overall survival, *C-index* Concordance index, *CI* confidence interval, *RFS* recurrence-free survival

In the training cohort, the time-dependent ROC analysis displayed that the CLIR score exhibited a distinctly better predictive value for OS than the Fong score (Wilcoxon matched-pair signed-rank *p* = 0.004) (Fig. 4a). The AUC of the CLIR score was significantly more extensive than the Fong score at a series of time points. For instance, the C-index of the 5-year OS probability forecast in the CLIR score was 0.721 (95% CI 0.691–0.751), which was significantly higher than that of the Fong score (C-index 0.640, 95% CI 0.607–0.673).

In the validation cohort (Fig. 4b), the time-dependent AUCs of the CLIR score model were significantly larger than those of the Fong score at a series of time points (*p *= 0.004). The 5-year OS C-index of the CLIR score was 0.665 (95% CI 0.634–0.696), which was also larger than that of the Fong score (0.603, 95% CI 0.570–0.636).

The CLIR score also exhibited better predictive values for RFS than the Fong score in the training (*p* = 0.031) and validation (*p* = 0.031) cohorts. The 2-year RFS C-index of the CLIR score was 0.728 (95% CI 0.699–0.757) in the training cohort and 0.640 (95% CI 0.608–0.672) in the validation cohort (Fig. 4c, d). These results suggested that the CLIR score was superior to the Fong score as it had better predictive discriminatory capacity for both OS and RFS.

### Survival Outcomes According to the Fong, CLIR, and Genetic And Morphological Evaluation (GAME) Scores in the Pooled Cohort and Subgroup Analysis

When applying the Fong and CLIR scores to the pooled cohort, the survival distributions showed the same trends as the training and validation cohorts (electronic supplementary Fig. S2). In the CRS model, the median OS for scores 0–5 was not reached, 100.0 months, 70.0 months, 45.0 months, 33.0 months, and 15.0 months, respectively, while in the CLIR model, the median OS in the corresponding scores was 134.0 months, 94.3 months, 43.0 months, 31.0 months, 18.7 months, and 13.0 months, respectively. The proportion of patients in the low-, intermediate-, and high-risk groups was 24.7%, 68.7%, and 6.6% for the Fong score, respectively, and 57.1%, 38.6%, and 4.3% for the CLIR score, respectively. The median OSs in the CRS and CLIR models were both 134.0 months for the low-risk group, 58.1 months and 39.9 months for the intermediate-risk group, and 29.0 months and 18.7 months for the high-risk group, respectively.

As for the GAME score, the survival curve distributions were not as good as the CLIR score (electronic supplementary Fig. S2). Median OS for scores 0–7 was not reached, 70.0 months, 63.6 months, 58.0 months, 44.0 months, 25.0 months, 38.6 months, and 10.0 months, respectively. Notably, the GAME score did not show adequate discrimination ability between the intermediate- and high-risk groups. Median OS was not reached in the low-risk group (score 0–1), 59.5 months in the intermediate-risk group (score 2–3), and 34.9 months in the high-risk group (score 4–7), respectively. The time-dependent ROC analysis demonstrated that the CLIR score exhibited a better predictive value for OS than the GAME score (*p* = 0.016) [electronic supplementary Fig. S3].

Besides, the CLIR score could better distinguish the intergroup survival of patients than the Fong score in the presence of extrahepatic diseases (electronic supplementary Fig. S4). Specifically, among patients with extrahepatic disease, the OS was distinguishable by different values in the CLIR score (*p* < 0.001), while the survival curves in the Fong score intersected (*p* = 0.117).

### Correlation Between Low-Density Lipoprotein–Cholesterol/High-Density Lipoprotein–Cholesterol Ratio and Clinical Characteristics

Next, we performed an exploratory analysis to investigate the correlation between the LHR and clinical characteristics. Our results showed that patients with elevated LHR (>2.9) had higher BMI (*p* = 0.016) and C-reaction protein levels (*p* < 0.001) [electronic supplementary Fig. S4]. We also observed that patients with CRLM >5 cm had a higher proportion of elevated LHR than patients with CRLM ≤5 cm (49.5% vs. 36.0%; *p* = 0.009) [electronic supplementary Table S1].

However, it was interesting to note that LHR did not vary with the majority of clinicopathology variables. First, LHR was not associated with preoperative serum biomarkers such as CEA levels. Second, LHR showed no statistical difference when stratified by demography characteristics (age and sex), primary tumor characteristics (tumor location, type or differentiation of pathology, T and N stage, and *KRAS* mutation), metastatic site characteristics (presence of extrahepatic disease, number of CRLMs, and Fong score), or systematic inflammation biomarkers (LDH levels, LNR, LMR, and lymphocyte-to-platelet ratio; *p*-values >0.05 for all) [electronic supplementary Table S1]. These findings suggested that high LHR may denote aggressive biology in a way that was independent of common clinicopathologic factors.

## Discussion

The current study proposed new insights into the association between systematic lipid, inflammation state, and CRLM resection. The results identified preoperative serum LHR and LDH levels as reliable and independent laboratory biomarkers for predicting the outcomes of complete CRLM resection. The newly developed CLIR score is an ideal prognostic signature that is inexpensive, simplified, clinically feasible, and independent of conventional classifications (i.e., time of occurrence of CRLM and tumor markers). Furthermore, the CLIR score demonstrated a better discrimination ability than the GAME score, which comprised *KRAS* mutation status (electronic supplementary Figs. S2 and S3). This finding is of great interest as LHR and LDH are readily available at a much lower cost than genomic markers.

**Fig. 5:**
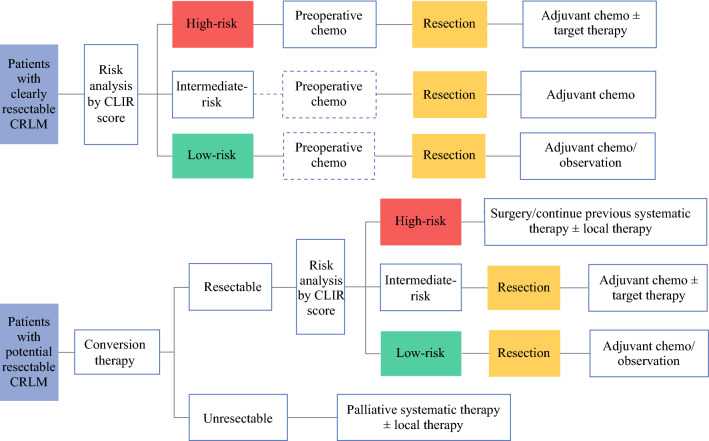
Therapeutic decision workflow for resectable or potentially resectable CRLM. *CRLM* colorectal liver metastasis, *CLIR* Circulating Lipid- and Inflammation-based Risk score, *chemo* chemotherapy

In the pooled cohort, the CLIR score classified nearly two-thirds of patients with very distinct behaviors: 57.1% with outstanding outcomes (score 0–1), as opposed to 4.3% with poor outcomes (score 4–5). Remarkably, the median OS of patients in the high-risk group was numerically more discriminative in the CLIR score than in the Fong score (18.7 months vs. 29.0 months). On the other hand, the CLIR score could identify a higher proportion of patients in the low-risk group (score 0–1) than the Fong score (57.1% vs. 24.7%), with the same OS of 134.0 months (electronic supplementary Fig. S2). Furthermore, the CLIR score could better distinguish survival than the Fong score in the presence of extrahepatic disease (electronic supplementary Fig. S4). Summing up the above, compared with the Fong score, the CLIR score could better define a portrait of the optimal candidates for CRLM resection with long-term survivals who might be suitable for less intensive perioperative chemotherapy regimens. The CLIR score also refined the selection of patients in whom hepatectomy could be less beneficial, while a multimodal therapy (systemic therapy or locoregional therapies such as ablation/radiotherapy/hepatic artery infusion chemotherapy) would be preferable. Therefore, the CLIR score might contribute to optimizing the combination of conversion therapy, neoadjuvant/adjuvant chemotherapy, and hepatectomy for CRLM patients (Fig. 5).

Our findings suggested that the preoperative LDL-C to HDL-C ratio measurement might inform the clinical decision making of CRLM resection. LDL-C was often called the ‘bad’ cholesterol, whereas HDL-C was termed the ‘good’ cholesterol.^51^ A higher LHR has been shown to predict heart attack, stroke, and atherosclerotic cardiovascular diseases.^52,53^ Increased cholesterol synthesis and uptake commonly characterize sustained cellular proliferation.^54,55^ Accumulating data have indicated the crucial role of endogenous cholesterol in the pathobiology of neoplasia, such as colorectal adenoma, breast cancer, and prostate cancer.^56–58^ Notably, decreased circulating HDL-C levels were identified as risk factors of gastrointestinal cancers.^59,60^ However, to the best of our knowledge, this present study is the first to address the impact of circulating lipids profile on predicting the long-term outcomes of complete CRLM resection.

Clinically, obesity is crudely estimated by the BMI (body mass/height^2^); however, available evidence suggests that BMI may have no significant prognostic relevance on CRLM resection.^61–63^ Patients with increased BMI even reportedly have prolonged OS after hepatectomy in various cancer types.^64^ One of the challenging aspects of using BMI is that lipid metabolism dysfunction does not exclusively occur in patients who are currently defined as obese.^65^ Approximately 50% of obese individuals remain metabolically healthy, while one-third of normal-weight individuals display metabolic ‘obesity’.^66–68^ Although elevated LHR was associated with higher BMI in the present study (electronic supplementary Fig. S5), BMI and other lipid markers were not robust predictive factors in multivariate analysis.

Adipocyte–tumor cell cross-talk is a vital process within the microenvironment that can promote tumor progression. Prior studies noted conflicting findings on the association of hepatic steatosis and recurrence following CRLM resection.^69^ Some studies outlined that non-alcoholic fatty liver disease (NAFLD) reduced the risk of recurrence,^70,71^ while others had opposite conclusions.^72–74^ Indeed, NAFLD was not a prognostic factor for CRLM resection in our dataset. Therefore, accurately characterizing the lipid metabolism state would likely require more precise assessments than BMI and NAFLD.

Cholesterol metabolism is regulated by a series of biosynthetic and transport mechanisms that rely on a continuous exchange between tissues and systemic circulation.^75^ A relationship between intracellular cholesterol content and cholesterol distribution in the plasma compartment has been previously detected.^76^ It is thus conceivable that the local and systemic environments are reprogramed together in individuals with poor adipose health, and alteration of cholesterol metabolism at the cellular level may entail changes in circulating cholesterol amounts. Therefore, assessing LHR derived from the peripheral blood may represent an appropriate method for defining the pathophysiologic consequences of cholesterol metabolism disorder.

In the present study, LHR was an independently significant predictor for OS. Of note, LHR was not associated with most clinicopathologic factors except for the maximum diameter of CRLM (electronic supplementary Table S1)*.* Furthermore, LHR was neither significantly related to tumor markers (CEA levels) nor systematic inflammation markers (LDH levels, lymphocyte-to-platelet ratio, LMR, and LNR; *p* > 0.05 for all) [electronic supplementary Fig. S5]. These observations may suggest that LHR denotes a mechanistic pathway that we did not investigate before.

In this study, we propose several hypotheses to explain why circulating lipid profile may confer prognostic information. (1) It was reported that dyslipidemia could alter the composition of energy balance-related host factors in the tumor site to provide a non-stop energy supply. Tumors that evolve within an obese microenvironment may exhibit ‘obesity addiction’, which depends on hypernutrition.^77^ (2) Circulating cholesterol metabolites could induce epithelial-to-mesenchymal transition (EMT), a cellular process associated with enhanced metastatic proficiency.^78^ Hence, it is convincible that enhanced cholesterol metabolism may create a favorable milieu for extrahepatic metastases growth. (3) Increased intracellular oxidative stress is involved in tumor development.^79^ HDL-C displays antioxidative activity and confers protection against oxidation of LDL-C. Conversely, low concentrations of HDL-C lead to more oxidized LDL-C, which is a cause of increased oxidative stress.^80,81^ (4) As adipose tissue outgrows its blood supply, hypoxia, adipocyte death, and ensuing adipose inflammation may occur.^41^ Adipose-associated inflammation fosters a tumor-supportive environment through local and systemic effects. By transforming the local landscape to closely resemble a healing wound, adipose inflammation may accelerate tumor growth.^82^ On the other hand, adipose inflammation and low-grade systemic inflammation are coupled, and each perpetuates the other.^83^ For instance, LDL-C can indirectly upregulate proinflammatory cytokines such as interleukin (IL)-1, IL-6, and tumor necrosis factor (TNF)-α in the TME, while downregulating the anti-inflammatory cytokines such as IL-10.^84,85^ Additionally, numerous cytokines and growth factors synthesized by adipocytes have a direct carcinogenic effect.^38–40,86^ Consistently, our study revealed that LHR correlates with the serum levels of C-reactive protein (electronic supplementary Fig. S5), which is the most representative marker reflecting systemic inflammatory responses in clinical settings.^87–89^ This ongoing inflammation can ultimately generate a pro-neoplastic environment by recruiting the immunosuppressive neutrophils to the TME.^31,90,91^ (5) HDL-C and LDL-C have been indicated as potential immunomodulators.^60,92^ Alterations in intracellular cholesterol homeostasis will affect immune cell function.^93^ LDL-C has been reported to enhance the function of immune-suppressive cells, such as myeloid-derived suppressor cells (MDSCs) and T-regulatory cells (Tregs), and inhibit the cytotoxic T-lymphocytes (CTLs), natural killer (NK) cells, and antigen-presenting cells such as dendritic cells (DCs).^94,95^ On the contrary, HDL-C involves converting the tumor-associated macrophages (TAMs) from a tumor-promoting phenotype (M2 type) to an anti-tumor phenotype (M1 type).^93,96^ Consequently, dyslipidemia contributes significantly to an immune-suppressive microenvironment within the tumor beds by regulating the activity of innate and adaptive immunity. Taken together, LHR could be more informative than single LDL-C or HDL-C, potentially providing a surrogate marker of a tumor-permissive microenvironment and worse tumor biology.

The metabolism of neoplastic cells is shifted toward high glucose uptake and lactate production. Elevated LDH levels are the product of enhanced glycolytic activity and tumor necrosis due to hypoxia.^97,98^ LDH serves as a negative prognostic biomarker not only because it is involved in metabolic adaptation but also because it blunts the tumor immunosurveillance by altering the TME.^36,99^ The current study reaffirms the reproducibility of LDH’s predictive value across differing populations. Considering the above evidence, we assumed that the CLIR score reflects the state of lipid metabolism and systemic inflammation, which both held value in assessing the immunity status.

Collectively, our findings provide a referable clinical basis for exploring the liver microenvironment changes in CRLM and suggest numerous avenues for follow-up studies. (1) Our study established the rationale for improving lipid metabolic health as a novel therapeutic target. Cholesterol-lowering medications such as statins might mitigate the pro-tumorigenic effects of dyslipidemia.^86^ (2) Agents such as metformin and vitamin D3 that target the intracellular energy balance pathways (e.g., P13K/Akt/mTOR) might hold great promise to reverse the obesity-driven aggressiveness.^100,101^ (3) In theory, nonsteroidal anti-inflammatory drugs such as aspirin could be beneficial in treating local and systemic inflammation.

Despite the promising results of this study, some limitations should be clarified. First, although the large cohort and independent external validation performed in this study may increase the reliability of our findings, the study is nonetheless limited by its retrospective nature. A long time span of patient inclusion could have induced some bias in OS analysis. Second, we did not propose a mechanistic understanding of why LDH and LHR could be associated with additional prognostic information; therefore, the question remains whether they were intermediate factors or true risk factors initiating specific detrimental pathways. Third, we did not have access to all medical files of the included subjects, therefore the exact influence of medications such as lipid-lowering drugs on our findings remains uncertain.

## Conclusions

The newly developed CLIR score demonstrated promising ability for predicting the long-term survival of CRC patients after complete hepatic resection, thereby signifying that lipid metabolism disorder and systemic inflammation could be potential targets in CRLM treatment that warrant further research.

## Supplementary Information

Below is the link to the electronic supplementary material.

Supplementary file 1 (PDF 583 KB)

Supplementary file 2 (DOCX 28 KB)

## Data Availability

The datasets used and/or analyzed during the current study are available from the corresponding author on reasonable request.
