# Supplementary material for: Circulating Lipid- and Inflammation-Based Risk (CLIR) Score: A Promising New Model for Predicting Outcomes in Complete Colorectal Liver Metastases Resection
Source: Ann Surg Oncol. 2022 Jan 4;29(7):4308–23. doi: 10.1245/s10434-021-11234-0 (PMC9174322; doi:10.1245/s10434-021-11234-0)
Supplement: Supplementary file 1 — (PDF 583 KB) [file 10434_2021_11234_MOESM1_ESM.pdf]

Supplementary Figure 1

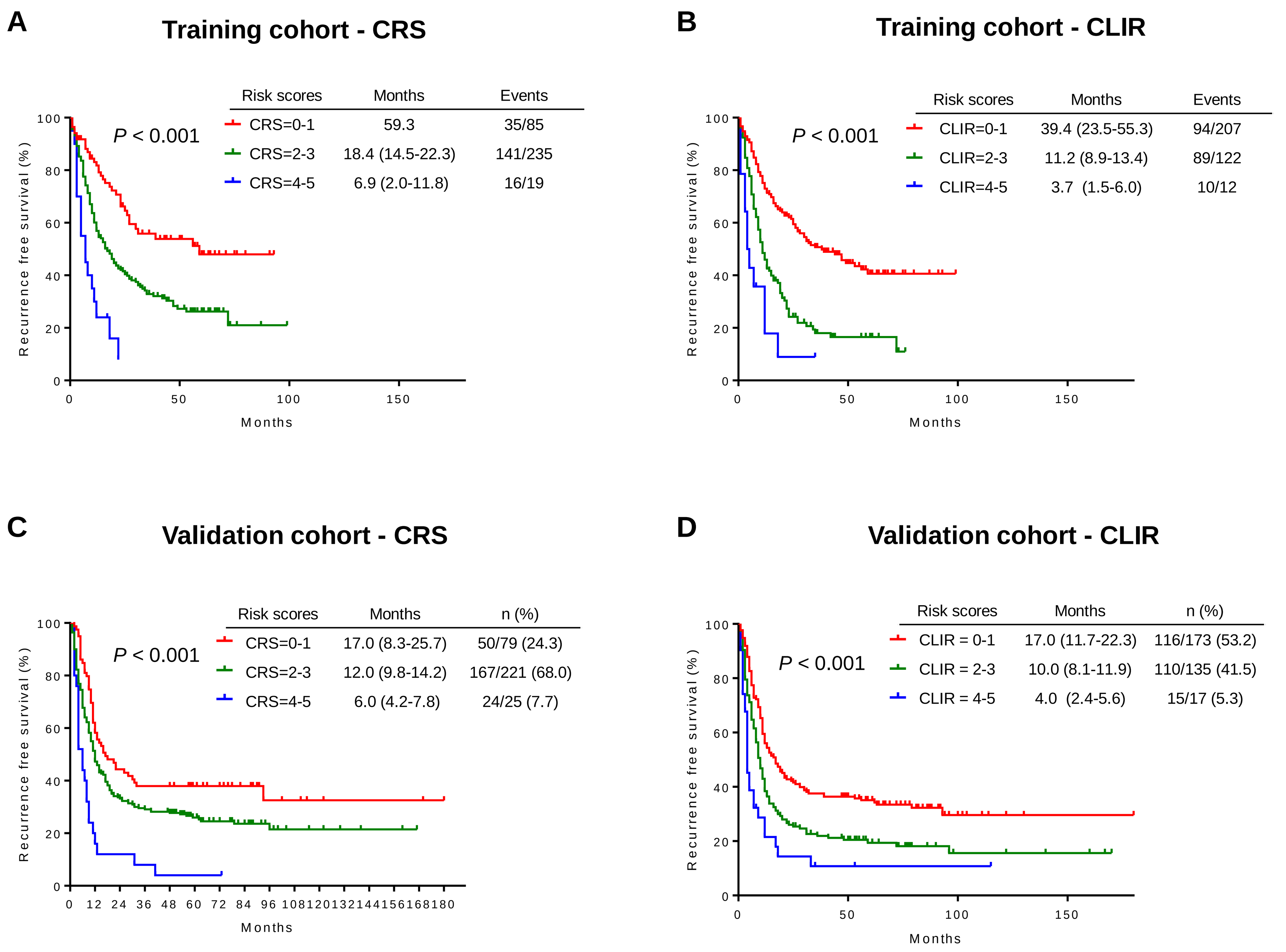

Supplementary Figure 1. Kaplan–Meier analysis of recurrence-free survival of patients stratified according to different risk groups. (A-B): Recurrence-free survival was stratified by different risk groups of CRS and CLIR in the training cohort. **(C-D)**: Recurrence-free survival was stratified by different risk groups of CRS and CLIR in the validation cohort; three categories: score 0-1, score 2-3, and score 4-5. Abbreviations: CRS, clinical risk score; CLIR, circulating lipid- and inflammation-based risk score.

Supplementary Figure 2

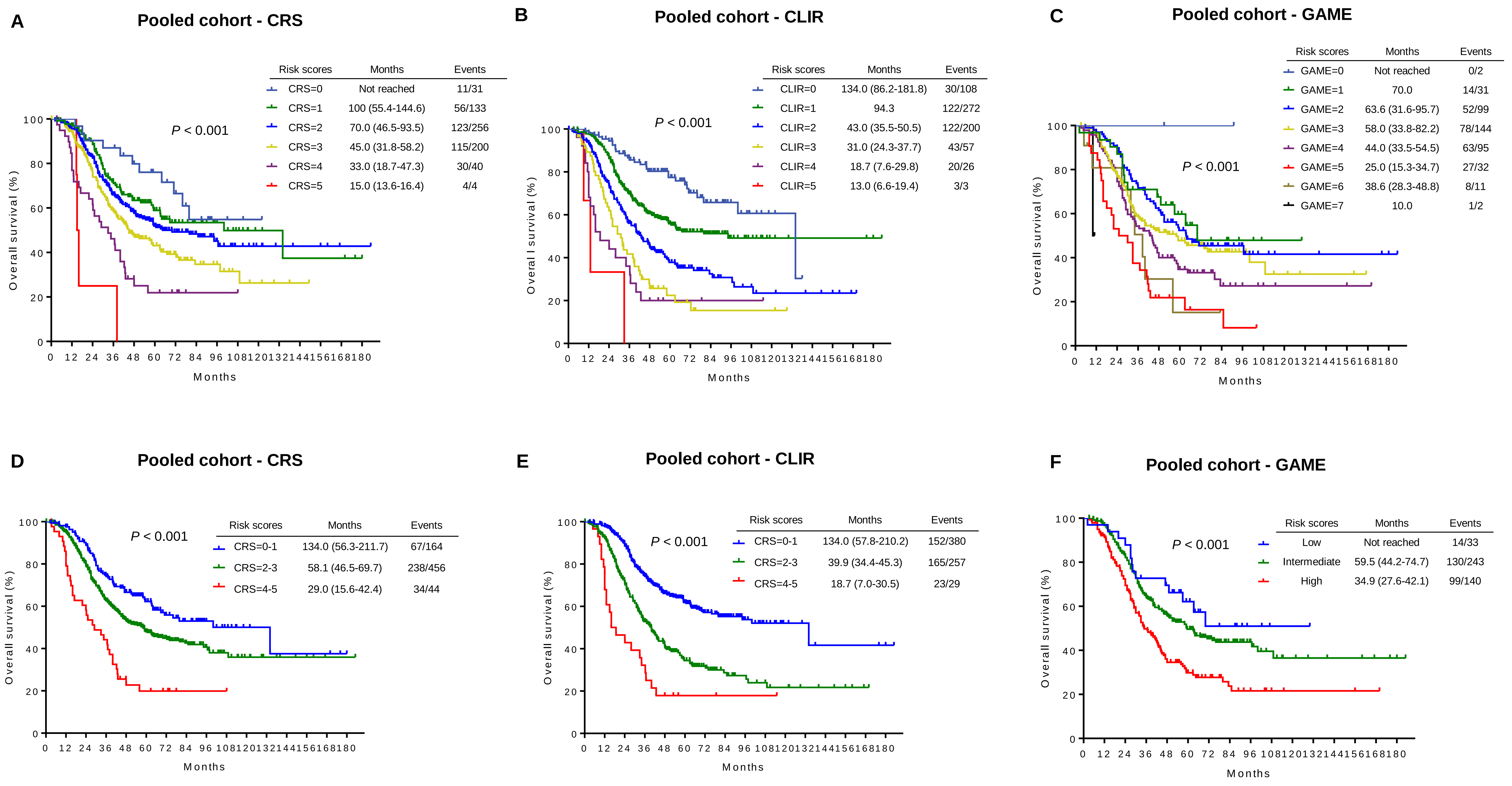

Supplementary Figure 2. Kaplan–Meier analysis of overall survival of patients in the pooled cohort stratified according to different scoring systems. **(A-C)**: Overall survival was stratified by different scores of CRS, CLIR, and GAME in the pooled cohort. **(D-F)**: Overall survival was stratified by different risk groups of CRS, CLIR, and GAME scores in the pooled cohort; three categories: score 0-1, score 2-3, and score 4-5.

Abbreviations: CRS, clinical risk score; CLIR, circulating lipid- and inflammation-based risk score; GAME, genetic and morphological evaluation score.

Supplementary Figure 3

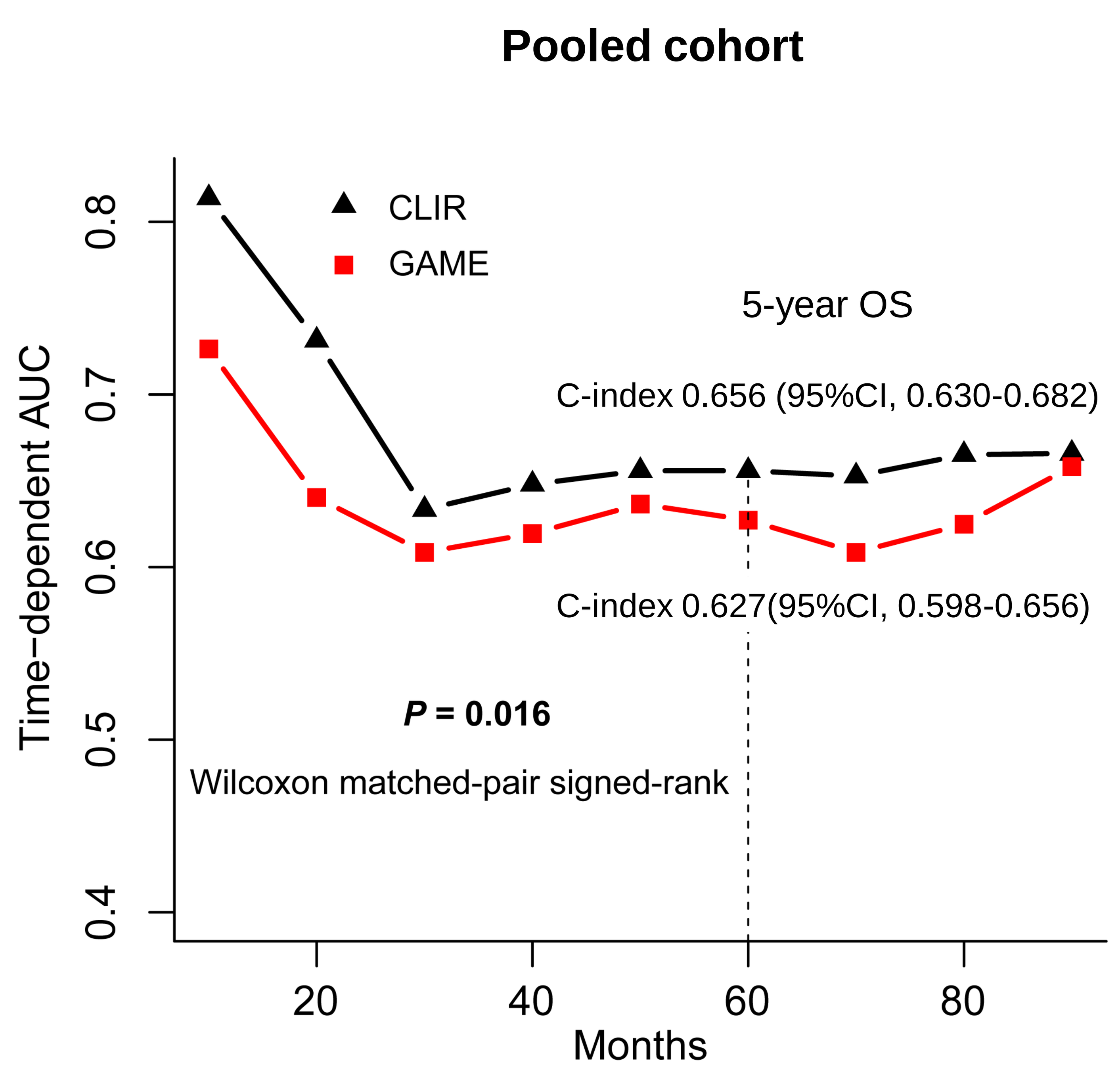

Supplementary Figure 3. Time-dependent AUCs of CLIR and GAME scores in the prediction of overall survival in the pooled cohort.

Abbreviations: CLIR, circulating lipid- and inflammation-based risk score; GAME, genetic and morphological evaluation socre; AUC, the area under the curve; OS, overall survival; C-index, index of concordance.

Supplementary Figure 4

A **Extrahepatic Disease - CRS**

B **Extrahepatic Disease - CLIR**

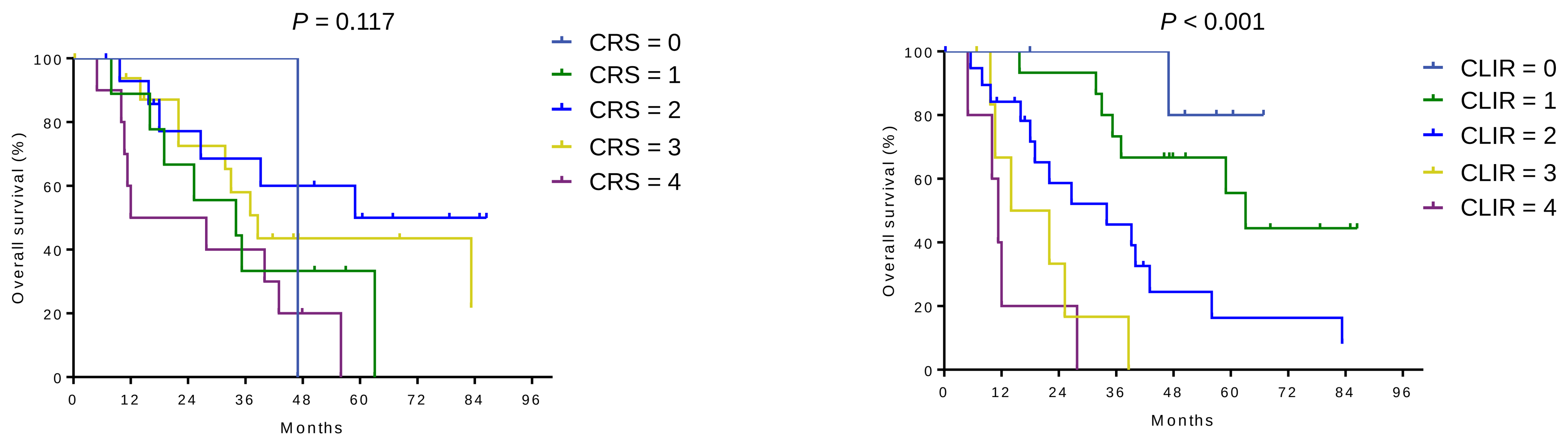

Supplementary Figure 4. Overall survival was stratified according to CRS and CLIR score estimated by Kaplan-Meier curves in patients with extrahepatic diseases.

Abbreviations: CLIR, circulating lipid- and inflammation-based risk score; CRS, clinical risk score.

Supplementary Figure 5

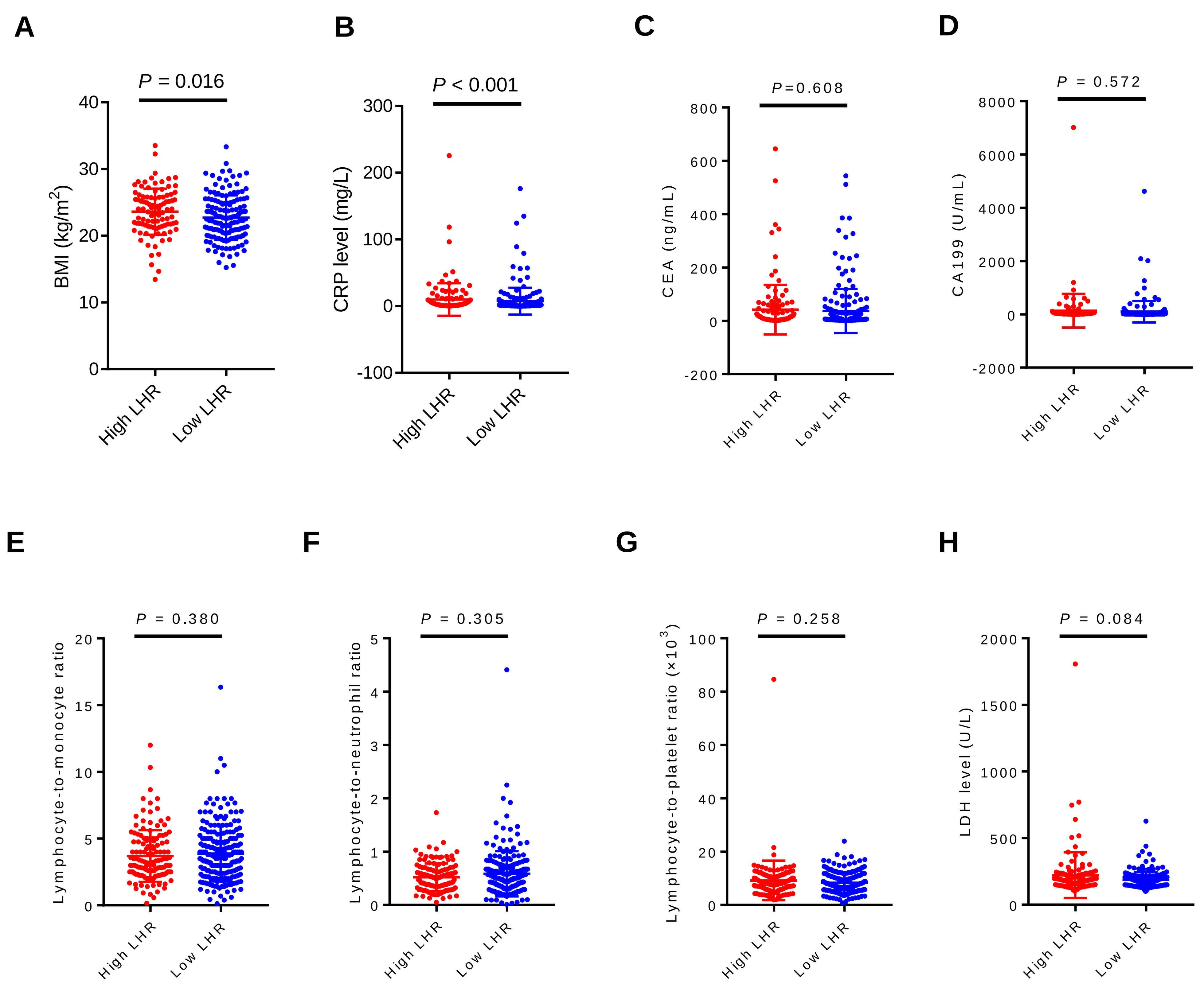

Supplementary Figure 5. Associations between preoperative serum LHR and other baseline clinical parameters in the training cohort. **(A)** LHR and BMI. **(B)** LHR and C-reactive protein levels. **(C)** LHR and CEA levels. **(D)** LHR and CA-199 levels. **(E)** LHR and lymphocyte-to-monocyte ratios. **(F)** LHR and lymphocyte-to-neutrophil ratios. **(G)** LHR and lymphocyte-to-platelet ratios. **(H)** LHR and LDH levels.

Abbreviations: LHR, low-density lipoprotein cholesterol-to-high-density lipoprotein cholesterol ratio; BMI, body mass index; CRP, C-reactive protein; LDH, lactate dehydrogenase.
