# Supplementary material for: Circulating Lipid- and Inflammation-Based Risk (CLIR) Score: A Promising New Model for Predicting Outcomes in Complete Colorectal Liver Metastases Resection
Source: Ann Surg Oncol. 2022 Jan 4;29(7):4308–23. doi: 10.1245/s10434-021-11234-0 (PMC9174322; doi:10.1245/s10434-021-11234-0)
Supplement: Supplementary file 2 — (DOCX 28 KB) [file 10434_2021_11234_MOESM2_ESM.docx]

Supplemental Tables for:

Circulating Lipid-and Inflammation-based Risk (CLIR) score: A promising new model for predicting outcomes in curative-intent colorectal liver metastases resection

De-Shen Wang et al.

**Table S1. Relation between patient clinical characteristics and LDL-to-HDL ratio in the pooled cohort**

| **Characteristics** | **LDL-C/HDL-C** | | | ***P* value** |
| --- | --- | --- | --- | --- |
|  | Under 2.9 ^a^, n (%) | Over 2.9, n (%) | |  |
| **Patient Characteristics** |  |  |  | |
| Age |  |  | 0.543 | |
| ≤65 | 332 (62.6) | 208 (37.4) |  | |
| >65 | 76 (59.4) | 52 (40.6) |  | |
| Gender |  |  | 0.129 | |
| Male | 265 (60.0) | 177 (40.0) |  | |
| Female | 148 (66.1) | 76 (33.9) |  | |
| **Primary tumor characteristics** |  |  |  | |
| Sidedness ^b^ |  |  | 0.421 | |
| Left-sided | 328 (61.3) | 207 (38.7) |  | |
| Right-sided  Location  Colon cancer  Rectal cancer  Pathology  Adenocarcinoma  Non-adenocarcinoma | 85 (65.4)  247 (62.8)  166 (61.0)  398 (62.6)  13 (46.4) | 45 (34.6)  146 (37.2)  106 (39.0)  238 (37.4)  15 (53.6) | 0.684  0.111 | |
| Differentiation |  |  | 0.599 | |
| Well/moderate | 343 (62.5) | 206 (37.5) |  | |
| Poor | 69 (59.5) | 47 (40.5) |  | |
| T stage |  |  | 0.135 | |
| Non-T4 | 308 (63.8) | 180 (36.2) |  | |
| T4 | 92 (57.1) | 73 (42.9) |  | |
| Lymph node metastases |  |  | 0.568 | |
| Absent | 167 (63.5) | 96 (36.5) |  | |
| Present | 246 (61.0) | 157 (39.0) |  | |
| *KRAS/BRAF* |  |  | 0.748 | |
| Wild type | 177 (63.9) | 100 (36.1) |  | |
| Mutation | 87 (62.1) | 53 (37.9) |  | |
| **CRLM characteristics** |  |  |  | |
| Largest metastasis site |  |  | **0.009*** | |
| ≤ 5cm | 358(64.0) | 199 (36.0) |  | |
| > 5cm | 55 (50.5) | 54 (49.5) |  | |
| Number of metastases |  |  | 0.297 | |
| 1 | 193 (64.3) | 111(35.7) |  | |
| > 1 | 220 (60.1) | 152 (39.9) |  | |
| Metastatic interval |  |  | 0.684 | |
| Metachronous | 165 (63.0) | 97 (37.0) |  | |
| Synchronous | 248 (61.4) | 156 (38.6) |  | |
| Extrahepatic disease |  |  | 0.133 | |
| Absent | 387 (62.8) | 229 (37.2) |  | |
| Present | 26 (52.0) | 24 (48.0) |  | |
| **CRS** |  |  | 0.225 | |
| 0-1 | 105 (64.0) | 59 (36.0) |  | |
| 2-3 | 284 (62.3) | 172 (37.7) |  | |
| 4-5 | 22 (50.0) | 22 (50.0) |  | |

^a^ 2.9 was the best cut-off for LDL-to-HDL ratio used in the current study.

^b^ Colorectal cancer arising in or proximal to the splenic flexure were defined as right-sided, and those arising distal to the splenic flexure were defined as left-sided.

Abbreviations: CRLM, colorectal liver metastases; CRS, Clinical Risk Score

* indicates statistical significance.

| **Table S2. Prognostic scoring systems** | | |
| --- | --- | --- |
| **Scoring system** | **Independent prognostic factors** | **Groups** |
| Fong score ^1^ | Node-positive primary (1 point)  Liver metastasis > 1 (1 point)  Metastasis size ≥ 5 cm (1 point)  Liver metastasis <1 year (1 point)  CEA > 200 ng/ml (1 point) | Low Risk (score 0-1)  Intermediate Risk (score 2-3)  High Risk (score 4-5) |
| GAME score ^2^ | Node-positive primary (1 point)  CEA ≥ 20 ng/ml (1 point)  *KRAS*-mutation (1 point)  Extrahepatic disease (2 points)  TBS between 3 and 8 (1 point) or 9 and over (2 points) | Low Risk (score 0-1)  Intermediate Risk (score 2-3)  High Risk (score 4-7) |
| CLIR score ^α^ | Node-positive primary (1 point)  Liver metastasis > 4 (1 point)  Metastasis size > 4.4 cm (1 point)  LDH > 260 U/L (1 point)  LDL-C-to-HDL-C ratio > 2.9 (1 points) | Low Risk (score 0-1)  Intermediate Risk (score 2-3)  High Risk (score 4-5) |
| ^α^ the CLIR score was the scoring model proposed in this paper; GAME score, Genetic and Morphological Evaluation score; TBS, tumor burden score; LDL-C, low-density lipoprotein cholesterol; HDL-C, high-density lipoprotein cholesterol. | | |

**References**

1. Fong Y, Fortner J, Sun R, et al: Clinical score for predicting recurrence after hepatic resection for metastatic colorectal cancer: analysis of 1001 consecutive cases. Annals of surgery 230:309-18; discussion 318-21, 1999

2. Margonis G, Sasaki K, Gholami S, et al: Genetic And Morphological Evaluation (GAME) score for patients with colorectal liver metastases. The British journal of surgery 105:1210-1220, 2018
